# Canonical and noncanonical autophagy: involvement in Parkinson’s disease

**DOI:** 10.3389/fcell.2025.1518991

**Published:** 2025-01-30

**Authors:** Maria Sakurai, Tomoki Kuwahara

**Affiliations:** Department of Neuropathology, Graduate School of Medicine, The University of Tokyo, Tokyo, Japan

**Keywords:** noncanonical autophagy, CASM, autophagy-related secretion, lysosome, Parkinson’s disease, α-synuclein, LRRK2

## Abstract

Autophagy is the major degradation process in cells and is involved in a variety of physiological and pathological functions. While macroautophagy, which employs a series of molecular cascades to form ATG8-coated double membrane autophagosomes for degradation, remains the well-known type of canonical autophagy, microautophagy and chaperon-mediated autophagy have also been characterized. On the other hand, recent studies have focused on the functions of autophagy proteins beyond intracellular degradation, including noncanonical autophagy, also known as the conjugation of ATG8 to single membranes (CASM), and autophagy-related extracellular secretion. In particular, CASM is unique in that it does not require autophagy upstream mechanisms, while the ATG8 conjugation system is involved in a manner different from canonical autophagy. There have been many reports on the involvement of these autophagy-related mechanisms in neurodegenerative diseases, with Parkinson’s disease (PD) receiving particular attention because of the important roles of several causative and risk genes, including LRRK2. In this review, we will summarize and discuss the contributions of canonical and noncanonical autophagy to cellular functions, with a special focus on the pathogenesis of PD.

## Introduction

Autophagy, the conserved cellular process discovered in the 1960s, plays an essential role in cell survival and homeostasis by internalizing various endogenous and exogenous materials into the lysosomes for degradation (Deter et al., 1967; Klionsky, 2007; Mizushima, 2007; Yamamoto et al., 2023). In particular, autophagy can selectively degrade misfolded proteins and damaged organelles in situations such as under stress. Autophagy has been classically classified into three major types: macroautophagy, microautophagy, and chaperone-mediated autophagy (CMA). Although these three pathways are largely different in terms of mechanism and function, some common molecules are involved (Mizushima and Komatsu, 2011; Watanabe et al., 2023; Yamamoto and Matsui, 2024). In macroautophagy, autophagosomes composed of double-membranes form (Baba et al., 1994) and are finally degraded by fusion with lysosomes. In microautophagy, vesicles incorporating cytoplasmic components form within lysosomes by membrane invagination, and their contents are degraded by rupture of the membrane (Schuck, 2020; Kuchitsu and Taguchi, 2024). In CMA, proteins are directly drawn into the lysosomes through the functions of the chaperone protein HSC70 and the lysosomal membrane protein LAMP2A (lysosomal-associated membrane protein 2 A) (Bourdenx et al., 2021). In recent years, however, it has become clear that the roles of autophagic mechanisms are not limited to the promotion of intracellular degradation. Of particular interest are the recently established noncanonical autophagy, also known as conjugation of ATG8 to single membranes (CASM) (Durgan et al., 2021; Durgan and Florey, 2022), and autophagy-related extracellular secretion (New and Thomas, 2019). Both of them uniquely require ATG proteins, especially those in the ATG8 conjugation system, as will be described later.

These autophagic and related mechanisms have been shown to be involved in a variety of diseases (Mizushima and Levine, 2020), including neurodegenerative diseases (Fleming et al., 2022; Nixon and Rubinsztein, 2024), immune system disorders (Deretic, 2021), and cancer (Debnath et al., 2023). Among these, Parkinson’s disease (PD), a major neurodegenerative disease along with Alzheimer’s disease, has accumulated such findings in the study of pathogenic mechanisms. PD is characterized pathologically by the dopaminergic neuron loss in the substantia nigra and the accumulation of Lewy bodies, the intraneuronal inclusions composed of fibrillated α-synuclein protein. Several PD-associated genes have been shown to be deeply related to the autophagic mechanisms, such as *SNCA* (*PARK1/4*), *PRKN* (*PARK2*), *PINK1* (*PARK6*), *LRRK2* (*PARK8*), *VPS35* (*PARK17*), and the PD risk gene *GBA1* (Blauwendraat et al., 2020; Ye et al., 2023). In particular, a variety of findings have accumulated on LRRK2 (leucine rich repeat kinase 2), which has been identified as a kinase that phosphorylates a subset of Rab GTPases (Komori and Kuwahara, 2023; Alessi and Pfeffer, 2024). Autophagy ultimately leads to degradation in lysosomes regardless of its type, and there have also been many reports on the relationship between PD and lysosomes (Abe and Kuwahara, 2021; Morris et al., 2024). For example, α-synuclein is at least partly degraded in lysosomes through the activity of the lysosomal enzymes including cathepsins (McGlinchey and Lee, 2015; Prieto Huarcaya et al., 2022), and decreased activities of glucocerebrosidase (GCase) (Balducci et al., 2007; Alcalay et al., 2015), cathepsin D (Parnetti et al., 2014; Kang et al., 2021), β-glucocerebrosidase (Parnetti et al., 2017), and α-gal (Alcalay et al., 2018; Wu et al., 2008) have been reported in cerebrospinal fluid (CSF), plasma or blood of sporadic PD patients. Mutation in the lysosomal membrane protein LIMP2 (lysosomal integral membrane protein 2) has been linked to PD (Michelakakis et al., 2012), and LIMP2 overexpression was shown to cause enhanced degradation of α-synuclein (Rothaug et al., 2014). The cytoplasmic aggregates of α-synuclein are thought to be related to neuronal loss, whether as its cause or result, and the involvement of autophagy has been repeatedly discussed.

**FIGURE 1:**
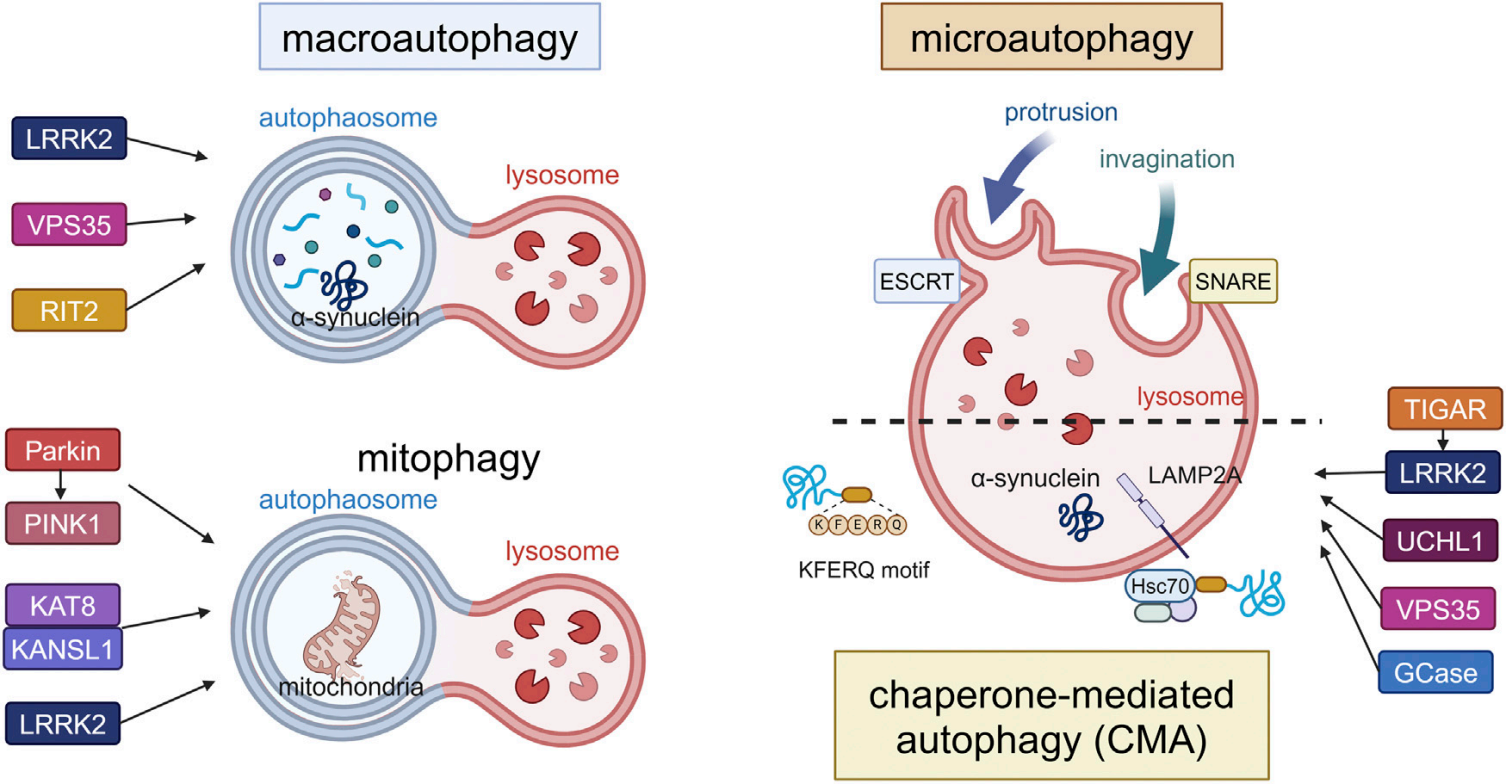
Canonical forms of autophagy and the involvement of PD-associated proteins. The three forms (macroautophagy, microautophagy, CMA) and mitophagy as a form of macroautophagy are schematically illustrated. In macroautophagy, double-membrane autophagosomes fuse with lysosomes to degrade proteins and damaged organelles such as mitochondria (mitophagy), while proteins are directly incorporated into lysosomes in microautophagy or CMA. LRRK2, VPS35 and RIT2 are involved in the upregulation of macroautophagy, while Parkin, PINK1, KAT8, KANSL1 and LRRK2 are involved in mitophagy. TIGAR, LRRK2, UCHL1, VPS35 and GCase are involved in CMA. α-Synuclein can be degraded by these pathways, possibly depending on its folding state. Abbreviations: ESCRT: endosomal sorting complex required for transport, GCase: β-glucocerebrosidase, KANSL1: KAT8 regulatory NSL complex subunit 1, KAT8: K (lysine) acetyltransferase 8, LAMP2A: lysosomal membrane protein 2A, LRRK2: leucine rich repeat kinase 2, PINK1: PTEN induced kinase 1, RIT2: Ras like without CAAX 2, SNARE: soluble N-ethylmaleimide-sensitive factor attachment protein receptors, TIGAR: TP53-induced glycolysis and apoptosis regulator, UCHL1: ubiquitin carboxy-terminal hydrolase L1, VPS35: vacuolar protein sorter-35. This figure was created in BioRender. Kuwahara, T. (2025) https://BioRender.com/f65q052.

In this review, we first overview the latest findings on the three classical autophagy machineries focusing on studies that have reported associations with PD (Figure 1). Then, we will also summarize and discuss recent knowledge on CASM and extracellular secretion that are regulated by autophagy-related mechanisms, since evidence is gradually accumulating on their relevance to PD.

### Macroautophagy and PD

Macroautophagy is well known to be triggered by starvation, and many proteins involved have been identified, as described next (Dikic and Elazar, 2018; Yamamoto and Matsui, 2024). In nutrient-rich conditions, ATG13 and ULK1/2 (unc-51 like autophagy activating kinase 1/2) are phosphorylated by the mammalian target of rapamycin complex 1 (mTORC1), whereas starvation leads to their separation from mTORC1 and resultant dephosphorylation. The autophagy initiation complex (the ULK complex) consisting of ULK1/2, FIP200, ATG13 and ATG101 is activated, and AMBRA1 (activating molecule in BECN1-regulated autophagy protein 1) phosphorylated by ULK1 binds to Beclin1, resulting in the activation of the class III phosphatidylinositol 3-kinase (PI3K) complex composed of VPS34, Beclin1, ATG14, AMBRA1, and p115. Activation of these complexes then initiates autophagosome formation. Membrane elongation leads to the lipidation of ubiquitin-like ATG8 with phosphatidylethanolamine (PE) via the involvement of ATG7, an E1-like enzyme, and ATG3, an E2-like enzyme (Taherbhoy et al., 2011; Fang et al., 2021; Liu et al., 2024). Also, the E3-like ATG12–ATG5-ATG16L1 complex forms via ATG7 and ATG10, the latter being another E2-like enzyme (Ichimura et al., 2000; Kuma et al., 2002). This ATG12–ATG5-ATG16L1 complex stabilizes ATG8 lipidation on target membranes (Mizushima et al., 1998; Hanada et al., 2007), while ATG4 promotes dissociation of ATG8 from PE and regulates the next round of autophagosome formation by promoting ATG8 recycling (Abreu et al., 2017). Autophagosomes fuse with lysosomes and are degraded by the action of SNAREs: STX17 (syntaxin 17), SNAP29 (synaptosome associated protein 29), and VAMP8 (vesicle associated membrane protein 8) (Itakura et al., 2012).

Impairment of macroautophagy has been suggested to contribute to neurodegeneration or its aggravation processes in PD (Johnson et al., 2019), and PD animal models and patients indeed show decreased macroautophagy. For example, PD model mice treated with 1-methyl-4-phenyl-1,2,3,6-tetrahydropyridine (MPTP) have been reported to show decreased lysosomal activity and accumulation of autophagosomes, while dopaminergic cell death was suppressed by treatment with rapamycin, an inducer of macroautophagy, or thioredoxin-1, a redox regulating protein (Dehay et al., 2010; Liu T. et al., 2023; Gu et al., 2024). In an ATG7 conditional knockout (cKO) mouse brain where autophagy is suppressed, a decrease in tyrosine hydroxylase (TH)-positive neurons and the aggregation of α-synuclein were observed (Ahmed et al., 2012). In addition, increased mTOR and decreased ATG7 were observed in the brains of dementia with Lewy bodies (DLB) patients and α-synuclein transgenic mice, with colocalization of α-synuclein and LC3 (Crews et al., 2010). Four novel variants of ATG7 were also identified in five sporadic PD patients, but not in the controls (Chen et al., 2013). On the other hand, it has been reported that extracellular secretion of α-synuclein with exosomes is enhanced in cells where macroautophagy is suppressed by the knockdown of ATG5, resulting in the suppression of α-synuclein-mediated cell death (Fussi et al., 2018). Despite this report, many of the studies suggest that decreased degradation of aggregated α-synuclein by inhibition of macroautophagy may lead to neurodegeneration.

On the other hand, some reports suggested that α-synuclein aggregation is the cause of macroautophagy failure. For example, macroautophagy is suppressed in α-synuclein overexpressing cells, and overexpression of RAB1A caused a rescuing effect through omegasome formation by ATG9 (Winslow et al., 2010). Microglia overexpressing α-synuclein showed enhanced phosphorylation of p38 and AKT and change of Akt/mTOR signaling, and the inhibition of autophagy in microglia led to dopaminergic neuron degeneration and behavioral abnormalities. Other reports have shown that activation of macroautophagy can rescue α-synuclein-mediated neuronal abnormalities (Tu et al., 2021). Aggregation of α-synuclein was reduced when autophagy was promoted by rapamycin in SH-SY5Y cells treated with α-synuclein pre-formed fibrils (PFF), but was increased when inhibited by chloroquine, a lysosome inhibitor (Gao et al., 2019). Overexpression of Beclin1 reduced intracellular aggregation of α-synuclein and rescued axon elongation (Spencer et al., 2009). Activation of the NLRP3 (NLR family pyrin domain containing 3) inflammasome occurred in MPTP-treated PD model mice, and the promotion of autophagic degradation of NLRP3 prevented neurodegeneration (Han et al., 2019). NLRP3 was also activated in dopamine neurons from parkin-depleted mice or PD patients and prevention of its activation was suppressed neurodegeneration (Panicker et al., 2022).

There are also several reports on the relationship between PD-associated genes other than α-synuclein and macroautophagy. As for LRRK2, it has been reported that expression levels of LRRK2 were increased in ATG5 or ATG7 cKO mice and KO MEF (mouse embyonic fibroblast) cells (Friedman et al., 2012). In neurons overexpressing G2019S mutant LRRK2 or G2019S knock-in mouse neurons, autophagosome trafficking was delayed because of mutant LRRK2-mediated recruitment of the motor adaptor JNS-interacting protein 4 (JIP4) (Boecker et al., 2021). As for another PD causative gene product VPS35, which is closely related to LRRK2, the PD-associated D620N mutation impaired its binding to the WASH complex and inhibited ATG9A trafficking, thereby suppressing macroautophagy (Zavodszky et al., 2014). More recent studies point to the involvement of RIT2 (Ras like without CAAX 2), a PD risk gene product and a small GTPase, in macroautophagy. RIT2 has been shown to activate LRRK2 (Obergasteiger et al., 2023), and vulnerability of RIT2-underexpressing dopaminergic neurons has been reported from analysis of *postmortem* brains of PD patients (Wang et al., 2024). RIT2 also regulates lysosomal function; RIT2 KO cells exhibited suppression of macroautophagy, while overexpression resisted α-synuclein aggregation (Gao et al., 2024). Since not only intracellular α-synuclein but also extracellular species are degraded by lysosomes after intracellular uptake (Sacino et al., 2017), the macroautophagy regulation shown by the above studies may influence overall α-synuclein dynamics, which may contribute to PD pathogenesis.

### Mitophagy and PD

Macroautophagy includes mechanisms that selectively degrade intracellular organelles, and among these, mitophagy has been strongly implicated in PD. Mitophagy acts to degrade damaged mitochondria and is essential for maintaining cell survival and homeostasis (Palikaras et al., 2018; Picca et al., 2023). There are two known pathways, ubiquitin-dependent and ubiquitin-independent (Khaminets et al., 2016), the latter employing protein-protein interaction motifs, ubiquitin-like modifiers, and sugar- or lipid-based signals. The ubiquitin-dependent pathway, on the other hand, employs two proteins responsible for autosomal recessive early-onset PD: phosphatase and tensin homologue (PTEN)-induced kinase (PINK1) and Parkin (Li et al., 2023; Narendra and Youle, 2024). Under normal conditions, PINK1 is transported to the mitochondrial inner membrane, where it is cleaved and degraded by proteases. However, when mitochondria are depolarized under stress, PINK1 accumulates on the mitochondrial surface and is activated by autophosphorylation to recruit Parkin, an E3 ubiquitin ligase, to the mitochondrial outer membrane. Parkin then ubiquitinates VDAC1 (voltage dependent anion channel 1) on the mitochondrial surface, which is essential for mitophagy(Geisler et al., 2010; Narendra et al., 2010; Ham et al., 2020). PINK1 also phosphorylates ubiquitin to activate Parkin and the phosphorylated ubiquitin chain further serves as a scaffold for adapter proteins to amplify mitophagy signals. Finally, the ubiquitin-binding adaptor p62/SQSTM1 mediates the recruitment of the LC3-localized phagophore to the ubiquitin-coated damaged mitochondria for degradation. Parkin also activates the ubiquitin-proteasome system to degrade mitochondrial outer membrane proteins, which is another critical process in mitophagy.

*Parkin* and *PINK1* mutations associated with early-onset PD are known as *PARK2* and *PARK6*, respectively (Hattori et al., 1998; Kitada et al., 1998; Leroy et al., 1998). In *Drosophila* harboring mutations in *PINK1* or *Parkin*, neuronal loss and muscle degeneration have been reported (Greene et al., 2003; Clark et al., 2006; Yang et al., 2006). Although *Parkin* KO in mice did not show signs of neurodegeneration, utilization of a mouse model that accumulates dysfunctional mitochondria revealed that *Parkin* KO in this model resulted in dopaminergic neuron degeneration with PD-like phenotypes (Pickrell et al., 2015). In *Pink1* KO mice, intestinal infection with Gram-negative bacteria caused activation of autoimmune system and PD-like phenotypes such as dopaminergic axonal loss and motor dysfunction (Matheoud et al., 2019). In humans, phosphorylated ubiquitin signaling as a downstream of the PINK1-Parkin pathway was elevated in the substantia nigra dopaminergic neurons of sporadic PD patients, whereas this was not evident in those of *PARK2* PD patients (Shiba-Fukushima et al., 2017). Phosphorylated ubiquitin in human brains was also shown to increase in an age- and Lewy body pathology-dependent manner, and colocalization of α-synuclein and phosphorylated ubiquitin in neurites was observed (Hou et al., 2018; Hou et al., 2023).

Of note, the role of PINK1/Parkin in mitophagy may somewhat differ between *in vitro* and *in vivo*; while these proteins are essential for mitophagy in cultured cells, mitophagy is still reported to occur in *Pink1* KO mouse brains and in PINK1/Parkin-deficient *Drosophila* (Lee J. J. et al., 2018; McWilliams et al., 2018). The possible reasons for this difference between *in vitro* and *in vivo* may be that more acute mitophagy stimuli are applied *in vitro* compared to chronic stimuli *in vivo*, and that other pathways independent of PINK1/Parkin compensate for mitophagy in rodents where the expression level of PINK1 is low (Han et al., 2023). Indeed, a set of other proteins have been known as mediators of Parkin-independent mitophagy (Onishi et al., 2021). Recently, a *PINK1* KO monkey model that exhibits neuronal loss in substantia nigra and cortex has been established (Yang et al., 2019; Yang et al., 2022). Since PINK1 expression is likely higher in primate brains than in rodents, studies using such models would be helpful to uncover the involvement of PINK1/Parkin-dependent mitophagy in PD.

As for the involvement of other proteins, cell-based knockdown screening of PD-associated genes identified KAT8 (lysine acetyltransferase 8) and KANSL1 (KAT8 Regulatory NSL Complex Subunit 1) as regulators of mitophagy. Both proteins interact with each other as the components of the non-specific lethal (NSL) complex, and their deficiency increased phosphorylated ubiquitin signaling in human iPSC-derived neurons (Soutar et al., 2022). In addition, a relationship between LRRK2 and mitophagy has also been suggested, as mitophagy was decreased in R1441C LRRK2 transgenic rats and iPSC-derived dopaminergic neurons from patients with LRRK2 R1441C mutation (Williamson et al., 2023). DJ-1, which is associated with *PARK7*, has been reported as an essential downstream of PINK1/parkin-mediated mitophagy (Imberechts et al., 2022). These findings suggest that disruption of mitochondrial homeostasis by impaired mitophagy may contribute to neurodegeneration.

### Microautophagy and PD

Microautophagy is another mode of autophagy reported in the 1980s (Marzella et al., 1981; Mortimore et al., 1983), where proteins are directly incorporated into lysosomes and undergo degradation. Like other types of autophagy, protein degradation by microautophagy is dependent on lysosomal pH and is inhibited by pH elevation by drugs such as chloroquine (Ahlberg and Glaumann, 1985; Ahlberg et al., 1985). There are at least two known pathways for microautophagy: the invagination pathway and the protrusion pathway (Mijaljica et al., 2011; Oku and Sakai, 2018; Wang L. et al., 2023). The invagination pathway requires the ESCRT complex, but the involvement of ATG proteins is limited and only the ATG8 and ATG12 conjugation systems are thought to be involved. On the other hand, the protrusion pathway requires ATG proteins and SNAREs, although the detailed molecular mechanism is still unclear. Microautophagy takes place on lysosomal and endosomal membranes and involves membrane engulfment, vesicle formation, and degradation. Endosomal microautophagy (eMI), like CMA described below, is triggered by the recognition of the KFERQ motif by HSC70 and works complementarily with CMA, although eMI selectively requires BAG6 (Krause et al., 2023). Currently, two types of eMI are believed to exist: ESCRT-dependent eMI and nSMase2-dependent eMI. Microautophagy is thought to be involved not only in protein degradation but also in the secretion of extracellular vesicles. The resultant regulation of cellular functions is diverse, and, for example, it has been reported that the cGAS-STING innate immune pathway is terminated through the degradation of STING by microautophagy (Kuchitsu et al., 2023).

Microautophagy has been less studied because it involves some of the same molecules as macroautophagy, and little has been reported on its effect on PD. Since several synaptic proteins have been reported to be degraded by eMI (Uytterhoeven et al., 2015), it is possible that α-synuclein undergoes degradation as well, and STK38-regulated microautophagy has been reported to prevent aging (Ogura et al., 2023), so further studies are warranted on the relationship between microautophagy and PD.

### CMA and PD

Chaperone-mediated autophagy (CMA) was first reported in the 1980s (Kaushik and Cuervo, 2018), and CMA substrates commonly harbor a unique sequence, the KFERQ motif (Dice, 1990). This motif is recognized by the chaperon HSC70 and the substrates are recruited to the lysosomes (Terlecky et al., 1992; Bandyopadhyay et al., 2008). Substrate-bound HSC70 then translocates into the lysosomal lumen *via* binding to the lysosomal membrane protein LAMP2A (Terlecky et al., 1992; Bandyopadhyay et al., 2008). CHIP and HSP40 further interact with HSC70 and facilitate the recruitment of substrates to the lysosomes (Agarraberes and Dice, 2001; Ferreira et al., 2013). This transport machinery to the lysosome is quite unique in that it does not involve dynamic membrane changes such as invagination or fusion, and multimerized LAMP2A forming a 700 kDa complex has been reported to mediate the translocation to the lumen (Bandyopadhyay et al., 2010). It should be noted, however, that the actual channel formation of LAMP2A has not been confirmed by means of structural analysis or *in vitro* reconstitution approaches (Neel et al., 2024).

CMA has been reported to be associated with several neurodegenerative diseases including PD, and α-synuclein has been demonstrated as a substrate of CMA (Cuervo et al., 2004; Vogiatzi et al., 2008; Liu Y. et al., 2023). The VKKDQ motif is present in α-synuclein, and its degradation is suppressed by lysosome inhibitors but not by macroautophagy inhibition. In addition, the expression level of LAMP2A was increased in the brains of α-synuclein transgenic mice, and the colocalization of α-synuclein with LAMP2A was also increased (Mak et al., 2010; Malkus and Ischiropoulos, 2012). Other studies have shown that the overexpression of LAMP2A reduced α-synuclein-induced neurotoxicity in cultured cells and in rat brains (Xilouri et al., 2013), while substantia nigra neuronal loss and behavioral abnormalities have been observed in rats following CMA suppression by inhibition of LAMP2A (Xilouri et al., 2016). In humans, decreased protein levels of LAMP2A and HSC70 were observed in the substantia nigra, amygdala and anterior cingulate cortex of PD patients (Alvarez-Erviti et al., 2010; Murphy et al., 2014), while there was no decrease in the levels of LAMP2B or LAMP2C in these patient brains (Murphy et al., 2015). In astrocytes derived from patients, the evidence of CMA abnormality was found, and pharmacological upregulation of CMA suppressed α-synuclein aggregation in co-cultured neurons (di Domenico et al., 2019). These findings suggest that suppression of CMA due to causes such as decreased LAMP2A levels may lead to PD.

Other PD-associated gene products have also received attention for their association with CMA. It has been reported that LRRK2 acts to inhibit the overall protein degradation by CMA (Orenstein et al., 2013). In the brains of R1441G LRRK2 knock-in mice, LAMP2A and HSC70 accumulated with age along with decreased CMA activity, and treatment with an activator of CMA was shown to reduce the amount of α-synuclein in the brain (Ho et al., 2020). In adipocytes of mice overexpressing TIGAR (TP53-induced glycolysis and apoptosis regulator), an inhibitor of the glycolytic pathway, increased levels of LRRK2 and RAB7B as well as LRRK2 activity-dependent suppression of CMA and macroautophagy were detected (Zhang et al., 2024). A *PARK5* gene product UCHL1 (ubiquitin carboxyl-terminal hydrolase L1) harboring PD-associated mutations has been reported to show aberrant interaction with CMA machinery components (Kabuta et al., 2008; Andersson et al., 2011). As for *PARK17* gene product VPS35 whose D620N mutation is associated with PD, LAMP2A-positive vesicles were reduced while α-synuclein-positive vesicles were increased in dopaminergic neurons from VPS35 knockout mice and D620N VPS35 overexpressing cells, due to enhanced degradation of LAMP2A in lysosomes (Tang et al., 2015). HDAC6 (histone deacetylase 6) has been suggested to regulate the pathologies of synucleinopathies including PD (Lemos and Stefanova, 2020), possibly by deacetylating HSP90 that regulates α-synuclein degradation by CMA (Du et al., 2021). Mutations in *GBA1*, the most common PD risk gene encoding the lysosomal enzyme glucocerebrosidase (GCase), have been shown to cause suppression of CMA as a result of mislocalization of mutant GCase to the lysosomal surface, thereby causing α-synuclein aggregation (Kuo et al., 2022). These reports collectively suggest the involvement of CMA in the degradation of α-synuclein, but as described above, the role of macroautophagy has also been suggested, and the contribution of each pathway would require further clarification.

### CASM and PD

**FIGURE 2:**
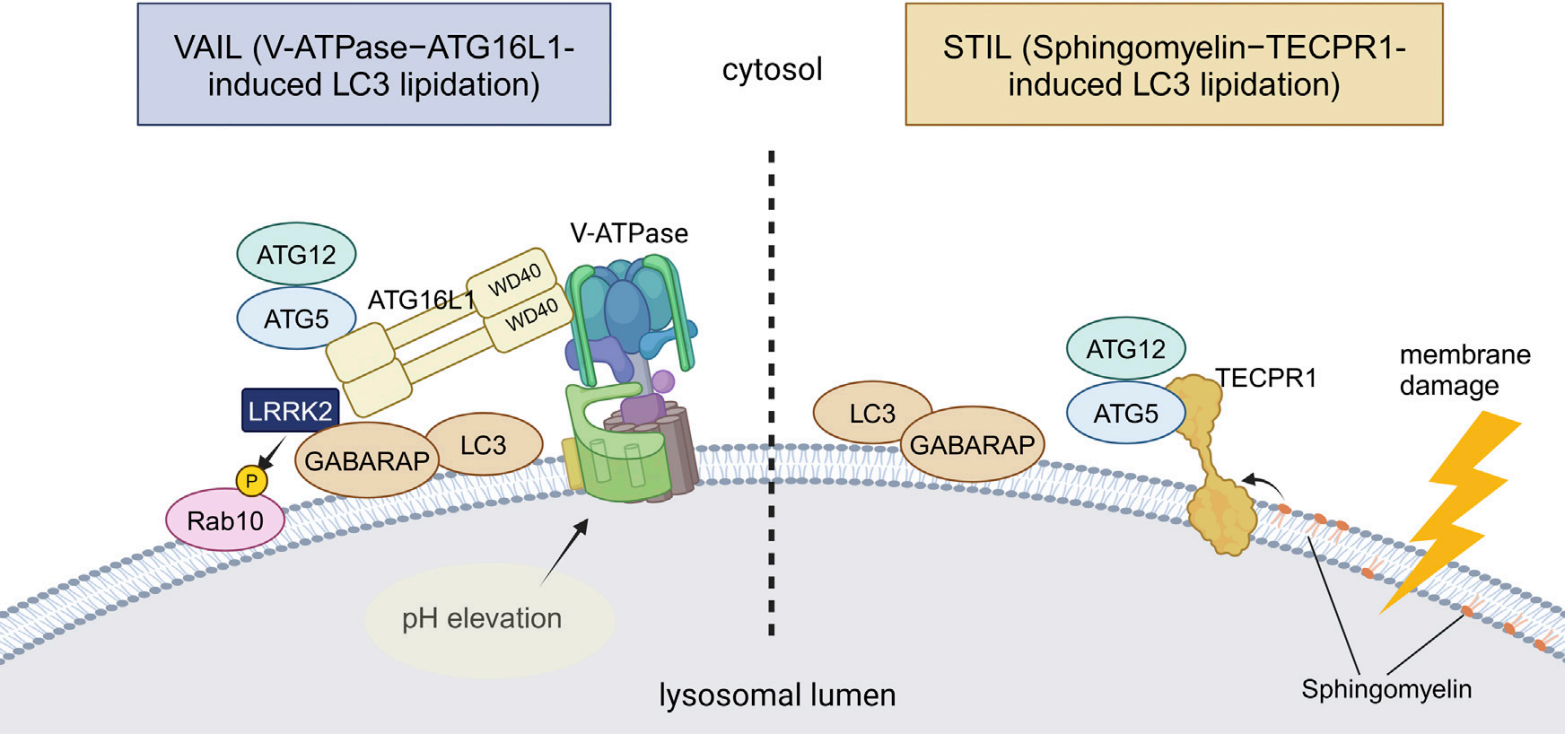
Two distinct mechanisms of CASM. VAIL and STIL, two recently reported mechanisms of CASM, are illustrated. In VAIL, the V-ATPase on lysosomes first forms a full complex upon lysosomal pH elevation. The WD40 domain of ATG16L1 then interacts with the V-ATPase, and the ATG12–ATG5-ATG16L1 complex recruits ATG8s onto lysosomes. In STIL, lysosomal membrane damage induces exposure of sphingomyelin and recruitment of TECPR1, which interacts with ATG12–ATG5 to recruit ATG8s onto lysosomes. Among PD-associated factors, the involvement of LRRK2 in VAIL has been suggested. Abbreviations: GABARAP: gamma-aminobutyric acid receptor-associated protein, TECPR1: tectonin beta-propeller repeat containing 1. This figure was created in BioRender. Kuwahara, T. (2025) https://BioRender.com/q74y182.

In macroautophagy, the conjugation of ubiquitin-like ATG8 proteins (termed ATG8ylation) occurs on double-membrane autophagosomes with the aid of the E3-like ATG12–ATG5-ATG16L1 complex. ATG8ylation has long been considered a specific marker and essential mechanism for autophagy. Recently, however, ATG8ylation has also been shown to occur on the single membranes of endolysosomes, which is collectively termed CASM (conjugation of ATG8 to single membranes) (Durgan and Florey, 2022; Wang et al., 2022). Currently, CASM is classified into two pathways: the VAIL (V-ATPase-ATG16L1-induced LC3 lipidation) pathway and the STIL (sphingomyelin-TECPR1-induced LC3 lipidation) pathway, each utilizing a different E3-like complex for ATG8 conjugation to single membranes (Deretic et al., 2024; Figueras-Novoa et al., 2024) (Figure 2).

VAIL, also known as the V-ATPase-ATG16L1 axis, is known to employ the ATG12–ATG5-ATG16L1 complex as E3 as in macroautophagy, but is more unique in that it requires the WD40 domain of ATG16L1, a domain not required for macroautophagy (Fletcher et al., 2018). Association of the V1 and V0 domains of V-ATPase, a proton pump on lysosomal membranes, triggers the recruitment of ATG16L1 onto the lysosomal membranes, resulting in the activation of VAIL (Hooper et al., 2022). Several endocytic events that have been named are included in VAIL, and LAP (LC3-associated phagocytosis) and LANDO (LC3-associated endocytosis) are known to be such specific types. LAP is characterized by LC3 lipidation on phagolysosomes that incorporate various pathogens or pathogen mimetics, such as the yeast cell wall preparation Zymosan (Sanjuan et al., 2007), influenza A virus (Beale et al., 2014; Fletcher et al., 2018; Wang et al., 2021), and *Helicobacter pylori* VacA toxin (Florey et al., 2015). Although the physiological roles of LAP are not necessarily clear, the acceleration of phagosomal maturation (Sanjuan et al., 2007) and antigen presentation (Ma et al., 2012; Romao et al., 2013; Fletcher et al., 2018) have been suggested. LANDO is characterized by the recruitment of LC3 onto endosomes that contain protein aggregates, although the physiological role of LANDO is still poorly defined (Peña-Martinez et al., 2022). In addition to these specific types, VAIL is known to be activated by various chemical compounds that act on lysosomes, such as lysosomotropic agents (e.g., chloroquine) (Florey et al., 2015), proton ionophores (e.g., monensin) (Florey et al., 2015), and the agonists of the lysosomal cation channel TRPML1/MCOLN1 (Goodwin et al., 2021). It has also been reported that VAIL is induced by activators of STING, which is involved in the innate immune response following the leakage of double-strand DNA into the cytoplasm (Gui et al., 2019; Fischer et al., 2020).

On the other hand, STIL, also known as the sphingomyelin-TECPR1 axis, is known to employ TECPR1 (tectonin beta-propeller repeat containing 1) instead of ATG16L1 as the component of functional E3 ligase complex containing ATG12–ATG5 (Boyle et al., 2023; Corkery et al., 2023; Kaur et al., 2023; Wang Y. et al., 2023). STIL is elicited upon lysosomal membrane damage caused by L-leucyl-L-leucine methyl ester (LLOMe) treatment or by infection with *Salmonella* or other bacteria. Lysosomal membrane damage leads to the cytosolic exposure of sphingomyelin, which in turn recruits TECPR1 by direct binding, resulting in ATG8 lipidation. However, STIL has only recently been reported, and its functions *in vivo* and relationship to disease are not yet clear.

For VAIL, possible links to PD or neurodegeneration have been suggested, albeit not many. We and others have shown that the VAIL pathway under CASM-causing stress induces the lysosomal recruitment and activation of PD-causative kinase LRRK2 (Bentley-DeSousa et al., 2025; Eguchi et al., 2024; Kuwahara and Iwatsubo, 2024). This activation causes the phosphorylation of its substrate RAB GTPases and facilitates the lysosomal stress responses, including the deflation of enlarged lysosomes and the exocytic release of lysosomal contents (Eguchi et al., 2024). Although papers from two groups have different views on the mechanism of LRRK2 recruitment/activation by VAIL, one report suggests that the ATG8 family member GABARAP recruits LRRK2 by direct binding (Bentley-DeSousa et al., 2025). Since aberrant activation of LRRK2 has been reported in idiopathic PD without LRRK2 mutations (Di Maio et al., 2018; Petropoulou-Vathi et al., 2022), the activation of the VAIL pathway may also be involved in the pathogenetic process of PD. There have been other reports that showed a link between VAIL and neurodegeneration, i.e., LANDO and Alzheimer’s disease (AD). LANDO suppressed amyloid-β (Aβ)-induced activation of microglia and deletion of LANDO components in microglia exacerbated neuronal death in AD model mice (Heckmann et al., 2019). Furthermore, the same group also reported that the deletion of WD40 domain of ATG16L1, essential for LANDO or VAIL, is sufficient for driving spontaneous AD pathology including deposition of endogenous Aβ and hyperphosphorylated tau, microgliosis and neurodegeneration in aged (2 years old) mice in the absence of AD protein overexpression (Heckmann et al., 2020) These findings suggest that CASM, especially the VAIL pathway, is involved in age-associated neurodegenerative processes, which should be further analyzed.

### Autophagy-related secretion and PD

**FIGURE 3:**
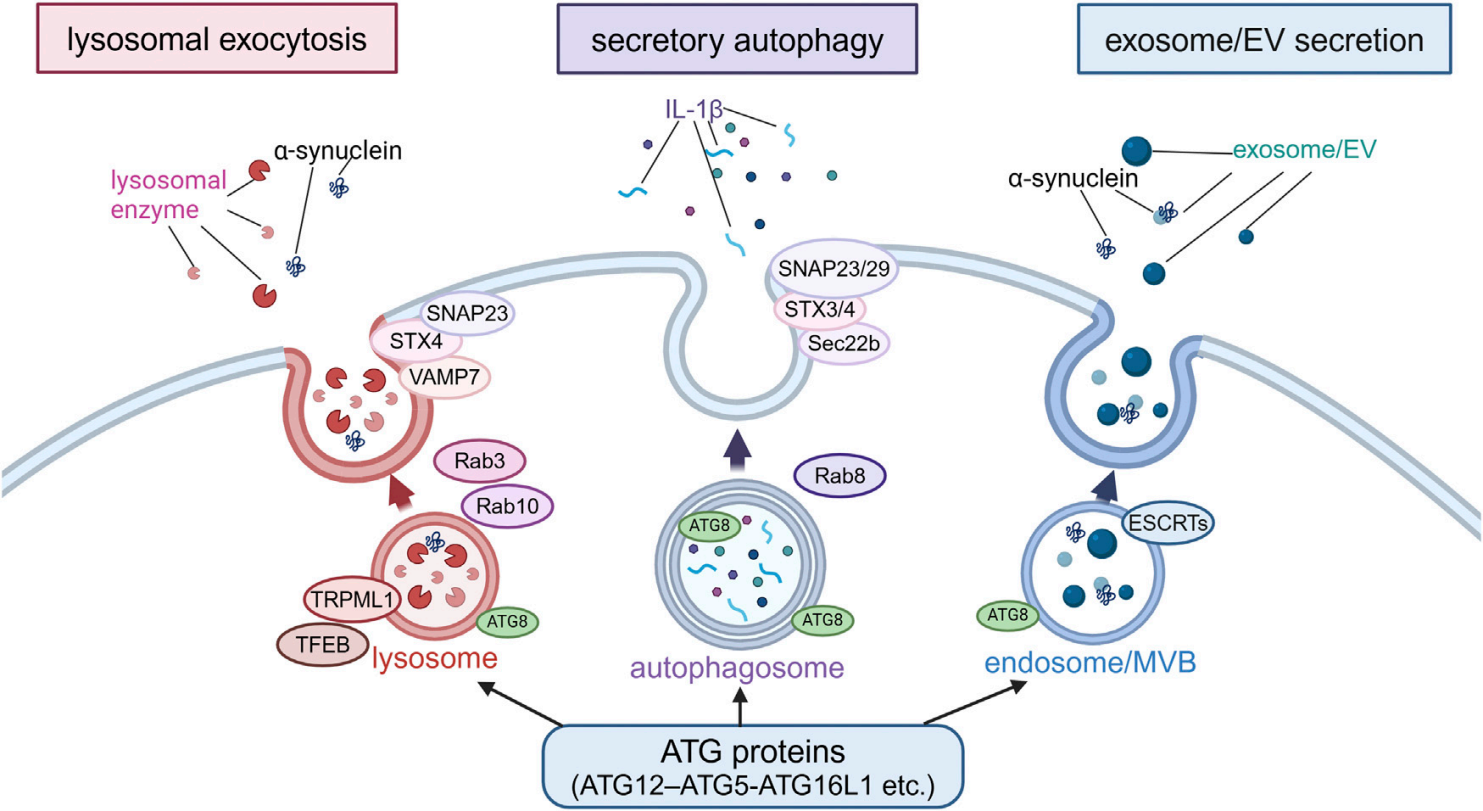
Extracellular secretion from endolysosomes regulated by autophagy-related proteins. Secretion modes regulated by non-autophagic functions of autophagy-related proteins were classified into three types based on the differences in membrane dynamics: lysosomal exocytosis, secretory autophagy and exosome/EV secretion. In lysosomal exocytosis, lysosomes fuse with the plasma membrane (PM) and lysosomal enzymes are released. In secretory autophagy, autophagosomes are responsible for the release of IL-1β and other proteins. In exosome/EV secretion, MBVs fuse with PM to release vesicles. SNAP23, STX, VAMP7, Rab3, Rab10, TRPML1 and TFEB are involved in lysosomal exocytosis, while SNAP23/29, STX3/4, Sec22b, and Rab8 are involved in secretory autophagy, and ESCRTs are involved in exosome/EV secretion. α-Synuclein has been suggested to be secreted in these modes. Abbreviations: EV: extracellular vesicle, MVB: multivesicular body, SNAP23: synaptosomal-associated protein 23, STX4: syntaxin 4, TFEB: transcription factor EB, TRPML1: transient receptor potential mucolipin 1, VAMP7: vesicle-associated membrane protein 7. This figure was created in BioRender. Kuwahara, T. (2025) https://BioRender.com/o81z371.

In recent years, it has gradually become known that the non-autophagic functions of ATG proteins are more diverse, including not only CASM but also extracellular secretion of endolysosomal compartments. Also, such secretory mechanisms have been thought to explain certain aspects of the neurodegenerative process in PD. There are various types of extracellular secretion involving ATG proteins or autophagy-related machineries, but the classification based on differences in membrane dynamics can be divided into three major types: lysosomal exocytosis, secretory autophagy, and exosomes/extracellular vesicles (EVs) secretion (Buratta et al., 2020). Although these three mechanisms are thought to overlap to some extent, a possible link to PD has been suggested for all three (Figure 3).

The first type, lysosomal exocytosis, is a process whereby lysosomes fuse with the plasma membrane and release their contents to the extracellular space, and is usually triggered by an increase in intracellular Ca^2+^ levels (Rodríguez et al., 1997; Martinez et al., 2000; Reddy et al., 2001). Mechanistically, one study has reported the crucial role of the ATG12–ATG5-ATG16L1 complex in this process (Tan et al., 2018), although this study shows the requirement of WD40 domain of ATG16L1, reminiscent of the regulation by CASM. Other autophagy-related proteins involved in this exocytosis process are transcription factor EB (TFEB) and the lysosomal Ca^2+^ channel TRPML1 (transient receptor potential mucolipin 1)/MCOLN1 (mucolipin TRP cation channel 1) (Medina et al., 2011). In addition, VAMP7, syntaxin-4 and synaptosome-associated protein of 23 kDa (SNAP23) work as SNARE proteins, and RAB3 and RAB10 work as regulators of membrane transport in lysosomal exocytosis (Rao et al., 2004; Encarnacao et al., 2016). Lysosomal exocytosis plays an important role in the regulation of diverse cellular functions, including the maintenance of plasma membrane integrity, extracellular matrix (ECM) remodeling, and defense against pathogens (Blott and Griffiths, 2002; Andrews, 2000; Tancini et al., 2020; Villeneuve et al., 2018; Neel et al., 2024). In addition, lysosomal exocytosis appears to contribute to some intercellular signaling by promoting the extracellular release of ATP (Zhang et al., 2007). In relation to neurodegenerative diseases, lysosomal exocytosis has been shown to mediate the extracellular release of TDP-43 and huntingtin, both of which contain the KFERQ motif and therefore possible substrates of CMA (Grochowska et al., 2023). In the context of PD, loss of ATP13A2/PARK9 has been shown to cause impaired Ca^2+^-induced lysosomal exocytosis and accumulation of α-synuclein in cultured human dopaminergic neurons (Tsunemi et al., 2019). Pathogenic α-synuclein was released from neurons *via* Ca^2+^-induced lysosomal exocytosis (Xie et al., 2022). Furthermore, although the mechanism may be different, α-synuclein aggregates can be secreted from endolysosomes via the mechanism called MAPS (misfolding-associated protein secretion), which employs the deubiquitinase USP19 and HSC70 co-chaperone DNAJC5/CSPα (Lee et al., 2016; Lee J. et al., 2018; Wu et al., 2023).

The second type of secretion, secretory autophagy, is originally considered as a mechanism whereby double-membrane autophagosomes directly fuse with the plasma membrane for the exocytic disposal of their contents. It should be noted, however, that there is no conclusive evidence for the fusion of autophagosomes with the plasma membrane, and it is also possible that autophagosomes are involved in steps preceding fusion (Dupont et al., 2011; Ejlerskov et al., 2013; Zhang et al., 2015; Neel et al., 2024). IL-1β is one of the few known proteins secreted via secretory autophagy (Dupont et al., 2011; Nakahira et al., 2011; Zhou et al., 2011). Amino acid starvation or treatment with NLRP3 inflammasome agonists has been shown to induce the extracellular secretion of IL-1β via the action of ATG5 and RAB8A (Dupont et al., 2011; Chang et al., 2024). Subsequent analyses have further identified Sec22b, syntaxin-3/4, and SNAP23/29 as regulators of secretory autophagy (Kimura et al., 2017; Chang et al., 2024). Also, as a specific type of secretory autophagy, SALI (secretory autophagy during lysosome inhibition) (Debnath and Leidal, 2022; Solvik et al., 2022) is reported to occur upon lysosome inhibition and MAD (migratory autolysosome disposal) (Sho et al., 2024) upon lysosome damage induction. The former occurs after autophagosomes fuse with late endosomes/multivesicular bodies (MVBs) to become amphisomes, and the latter occurs after they fuse with lysosomes to become autolysosomes. Given that these are not secretions from autophagosomes and that a set of ATG proteins are commonly involved, there may be some overlap with other secretion mechanisms including exosomal secretion and lysosomal exocytosis. In relation to PD, one study has shown that the extracellular secretion of α-synuclein is mediated by exophagy, a mode of secretion from an autophagy intermediate produced by impaired autophagosome-lysosome fusion, similar to secretory autophagy, and that this secretion is enhanced by overexpression of the tubulin polymerization-promoting protein (TPPP/p25α) (Ejlerskov et al., 2013). Another recent study has shown that neuronal activity-dependent enhancement of α-synuclein release is mediated by secretory autophagy (Nakamura et al., 2024). However, since these and other papers that suggest the involvement of secretory autophagy often show the secretion with exosomes/EVs, it may be difficult to make a clear distinction from the mechanism described next.

The third type of secretion involving autophagy-related machineries is mediated by exosomes or EVs, the former typically defined as vesicles 50–150 nm in diameter (Welsh et al., 2024). Exosomal secretion occurs when the endosomal membrane is invaginated to form intraluminal vesicles (ILVs), which are then secreted from the cell instead of being degraded in lysosomes. There are at least two pathways for exosome biogenesis: ESCRT-dependent and ESCRT-independent pathways (Catalano and O'Driscoll, 2020). Although the exact site of action of ATG proteins is not necessarily clear, one study has reported that ATG5 and ATG16L1 de-acidify MVBs via dissociation of the V_1_V_0_-ATPase to induce exosome release (Guo et al., 2017). Several studies have suggested a link to PD mechanisms; for example, inhibition of autophagy by treatment with bafilomycin A_1_ or chloroquine has been shown to enhance α-synuclein secretion via EVs harboring a hybrid autophagosome-exosome-like phenotype (Minakaki et al., 2018). TPPP/P25α-mediated exophagy of α-synuclein was accompanied by exosome secretion (Ejlerskov et al., 2013), and neuronal activity-mediated secretion of α-synuclein was found in both exosome associated and free forms (Nakamura et al., 2024) in the studies involving secretory autophagy described above as well. Another study has shown that extracellular secretion of α-synuclein as well as its propagation is enhanced when autophagy is suppressed by S-nitrosylation of p62 (Oh et al., 2022). In our study, treatment of microglia incorporating α-synuclein fibrils with lysosomotropic agents resulted in the secretion of insoluble α-synuclein, which was dependent on Rab10 phosphorylation by LRRK2 (Abe et al., 2024). This secretion was accompanied by the release of lysosomal luminal proteins, and CASM was responsible for the release by mediating LRRK2 activation (Eguchi et al., 2018; Eguchi et al., 2024). These findings collectively suggest that the extracellular secretion of endolysosomal contents, which is regulated by non-autophagic functions of autophagy-related proteins, may be involved in the pathomechanism of PD.

## Discussion and future perspectives

As reviewed thus far, recent studies have uncovered various forms of autophagy and related mechanisms as well as their association with PD (summarized in Table 1). Importantly, some autophagy-related mechanisms, especially the ATG8 conjugation system, play additional roles other than intracellular degradation (Nakamura et al., 2020; Wang et al., 2021; Wang et al., 2022). Therefore, the previously reported relationships between PD and autophagy mechanisms would need to be reconsidered, incorporating the latest information, to determine whether the findings really imply the involvement of intracellular degradation in the disease mechanisms. For example, when elevated LC3 lipidation is detected in PD or its model animals/cells, one must consider the possibility that CASM is activated, not simply that macroautophagy is activated. There are also two types of CASM, VAIL and STIL (Deretic et al., 2024; Figueras-Novoa et al., 2024), and such classifications may increase further in the future.

**TABLE 1:** Selected list of evidence suggesting a link between the autophagic pathway and PD. α-syn: α-synuclein, cKO: conditional knockout, DA: dopaminergic, DLB: dementia with Lewy bodies, KD: knockdown, MPTP: 1-methyl-4-phenyl-1,2,3,6-tetrahydropyridine, OE: overexpression, p-Ub: phosphorylated ubiquitin, Tg: transgenic, TH: tyrosine hydroxylase, Trx: thioredoxin-1, ↑: increase, ↓: decrease.

Reported phenotypes	Analyzed systems/models	References
Macroautophagy		
autophagosome accumulation ↑	MPTP-treated mice	Dehay et al. (2010)
TH-positive neurons ↓/α-syn accumulation ↑	MPTP-treated mice	Liu et al. (2023a)
TH-positive neurons ↑/α-syn clearance ↑	Trx-1 OE in MTPT-treated mice	Gu et al. (2024)
TH-positive neurons ↓/α-syn accumulation ↑	ATG7 cKO mice	Ahmed et al. (2012)
mTOR ↑/ATG7 ↓	DLB brains and α-syn Tg mice	Crews et al. (2010)
α-syn extracellular secretion ↑	ATG5 KD cells	Fussi et al. (2018)
Omegasome formation ↑	Rab1A and α-syn OE neuroblastoma	Winslow et al. (2010)
α-syn-mediated autophagy inhibition ↓	BV-2 cells and microglia with Akt/mTOR suppression	Tu et al. (2021)
α-syn accumulation ↓	Rapamycin-treated SH-SY5Y cells	Gao et al. (2019)
α-syn accumulation ↓	Beclin1 OE B103 cells	Spencer et al. (2009)
Neurodegeneration by autophagic degradation of NLRP3 ↓	BV2 cells and microglia	Han et al. (2019)
LRRK2 expression ↑	ATG5 or ATG7 cKO mice	Friedman et al. (2012)
autophagosome trafficking ↓	LRRK2 mutant neurons	Boecker et al. (2021)
ATG9A trafficking ↓	VPS35 mutant HeLa and SH-SY5Y cells	Zavodszky et al. (2014)
autophagy ↓	RIT2 KO SH-SY5Y cells	Gao et al. (2024)
Mitophagy ↓	Parkin KO *Drosophila*	Greene et al. (2003)
Mitophagy ↓	PINK1 mutant/KD *Drosophila*	Clark et al. (2006), Yang et al. (2006)
PD-like phenotype	Parkin KO mice	Pickrell et al. (2015)
p-Ub signaling ↑	PD dopaminergic neurons	Shiba-Fukushima et al. (2017)
p-Ub structures ↑	Lewy body disease brains	Hou et al. (2018)
p-Ub structures ↑	DLB brains with SNCA mutations/multiplications	Hou et al. (2023)
PINK1-dependent mitophagy ↓	KAT8, KANSL1 KD iNeurons	Soutar et al. (2022)
Mitophagy ↓	LRRK2 mutant rats and iPS-neurons	Williamson et al. (2023)
Mitophagy ↓	DJ-1-deficient fibroblasts and neurons	Imberechts et al. (2022)
CMA		
LAMP2 expression ↑	α-syn Tg mice	Mak et al. (2010)
LAMP2 expression ↑	α-syn mutant mice	Malkus and Ischiropoulos (2012)
α-syn-induced neurotoxicity ↓	LAMP2A OE primary neurons	Xilouri et al. (2013)
LAMP2A expression ↓	PD brains	Alvarez-Erviti et al. (2010), Murphy et al. (2014)
α-syn aggregation ↑	LAMP2A KD astrocytes	di Domenico et al. (2019)
CMA ↓	LRRK2 OE HEK293 cells	Orenstein et al. (2013)
α-syn aggregation ↓	LRRK2 mutant mice	Ho et al. (2020)
CMA ↓	TIGAR OE adipocytes	Zhang et al. (2024)
CMA ↓	VPS35 KO mice, mutant DA neurons	Tang et al. (2015)
CMA ↓	HDAC6 inhibitor-treated mice	Du et al. (2021)
CMA ↓	GBA1 mutant NIH3T3 cells	Kuo et al. (2022)
CASM		
activation of LRRK2 by VAIL pathway under lysosomal stress	RAW264.7 cells	Eguchi et al. (2024)
activation of LRRK2 by VAIL pathway	RAW264.7 cells	Bentley-DeSousa et al. (2025)
Autophagy-related secretion		
lysosomal exocytosis ↓/α-syn accumulation ↑	ATP13A2 mutant DA neurons	Tsunemi et al. (2019)
SNARE-dependent α-syn secretion	Α53Τ α-syn Tg mice	Xie et al. (2022)
α-syn secretion by late endosomes (independent of CMA) ↑	USP19 OE COS-7 cells	Lee et al. (2016), Lee et al. (2018a)
α-syn secretion ↑	DNAJC OE HEK293T cells	Wu et al. (2023)
α-syn secretion ↑	TPPP OE PC12 cells	Ejlerskov et al. (2013)
lysosomal secretion under lysosomal stress ↓	LRRK2, ATG5, ATG16L1 KD RAW264.7 cells	Eguchi et al. (2018), Eguchi et al. (2024)
α-syn secretion under lysosomal stress ↓	LRRK2 KD microglial cells	Abe et al. (2024)

In addition, the three cellular activities described so far—intracellular degradation, CASM, and extracellular secretion—are seemingly distinct events, but they do not necessarily function independently; rather, they are assumed to work in coordination around the lysosome. In particular, when lysosomal pH is elevated due to some dysfunction of lysosomes, autophagic flux is inhibited and CASM is induced, while extracellular release from endolysosomes also occurs (Bentley-DeSousa et al., 2025; Eguchi et al., 2024). Such lysosomal dysfunction is generally known to be accelerated by aging, as accumulation of intralysosomal granules such as lipofuscin is often observed in aging tissues (Goyal, 1982; Gray and Woulfe, 2005). Since PD is an age-associated disorder, lysosomal dysfunction is likely to be involved in the pathomechanism, and lysosomal activation would be one of the therapeutic strategies.

However, lysosomal activation involves complicated molecular mechanisms as described above, and in terms of drug discovery, it is important to determine which mechanism to activate and how to activate it. Therefore, further studies on autophagy and its related mechanisms are warranted. Additionally, the application to PD therapy requires model animals and cells that faithfully reflect the pathogenesis of PD, but unfortunately, these model systems are still under development. For example, although cellular models that show significant deposition of α-synuclein aggregates have been developed, would it be therapeutic if the deposition were reduced by the activation of autophagy or related pathways? A well supported view is that PD pathology develops via extracellular release of α-synuclein aggregates and their cell-to-cell propagation. If a decrease in intracellular accumulation of α-synuclein promotes its extracellular release, the modulatory effect on the pathogenesis needs to be carefully discussed. Ultimately, it will be essential to develop and use models that can comprehensively assess α-synuclein accumulation and neurodegeneration throughout tissues.

In summary, it is desirable to promote a comprehensive understanding of the autophagy-lysosome system and to further improve models of PD, and the combination of these findings and technologies is expected to lead to novel therapeutic strategies for PD.
